# *Tinea capitis* in children: changes in the epidemiological profile over 22-years of study^☆^

**DOI:** 10.1016/j.abd.2026.501476

**Published:** 2026-10-28

**Authors:** Esther Alves de Araujo Nunes, Mariana Aparecida Pasa Morgan, Beatriz Carvalho, Alon Coutinho Nascimento, Talita Sana Valério Fontana, Marjorie Uber, Kerstin Taniguchi Abagge, Renata Robl Imoto, Vânia Oliveira Carvalho

**Affiliations:** a Pediatric Dermatology Division, Department of Pediatrics, Universidade Federal do Paraná, Curitiba, PR, Brazil; b Postgraduate Program in Child and Adolescent Health, Faculdade de Medicina de Ribeirão Preto, Universidade de São Paulo, Ribeirão Preto, SP, Brazil; c Ceres Faculty, FACERES Medical School, São José do Rio Preto, Brazil; d Sector of Health Sciences, Faculty of Medicine, Universidade Federal do Paraná, Curitiba, PR, Brazil

Dear Editor,

*Tinea capitis* in children involves dermatophyte invasion of hair follicles and the scalp, manifesting as alopecia with scaling and broken hairs, or an inflammatory *kerion celsi.*^1^, ^2^ The causative agents belong to the genera *Microsporum* and *Trichophyton*, with a prevalence that varies geographically according to environmental and socioeconomic factors.^3^ Transmission occurs via anthropophilic (*Trichophyton tonsurans*), zoophilic (*Microsporum canis*), or geophilic routes.^1^, ^2^, ^3^ Shifts in dermatophyte distribution, driven by migration and lifestyle changes, have been observed. While *Microsporum canis* remains the leading global cause, some regions now show a predominance of *Trichophyton* species.^3^ Agent identification is critical for therapeutic success: *Microsporum canis* shows higher cure rates with griseofulvin, whereas *Trichophyton* infections respond better to terbinafine.^3^, ^4^ Furthermore, controlling *Microsporum canis* transmission requires treating animal carriers.^5^

In Brazil, epidemiological data remain scarce. This study aims to identify the causative agents of *tinea capitis* and their treatment response over time in a tertiary hospital in Southern Brazil.

This retrospective analytical study included children diagnosed with *tinea capitis* at a tertiary hospital in Southern Brazil (2022-2024). Approved by the Institutional Ethics Committee (CAAE 73519423.1.0000.0096), data were extracted from medical records, direct microscopy, and fungal culture. Clinical photographs were reviewed.

Patients up to 16-years of age diagnosed with *tinea capitis* were included. The epidemiological and clinical variables are summarized in Table 1. Inclusion criteria comprised typical clinical presentations, such as alopecia with scaling and broken hairs and *kerion celsi* forms, confirmed by positive fungal culture or, in culture-negative cases, a documented good response to antifungal treatment.

**Table 1:** Characteristics of patients with tinea capitis: demographic, clinical, mycological, and therapeutic data.

Characteristics	n = 130; n (%)
Sex	
Male	82 (63.1%)
Female	48 (36.9%)
Median age (months)	60 (2‒190)
Age group	
Infant	12 (9.2%)
Preschool-aged	43 (33.1%)
School-aged	67 (51.5%)
Adolescent	8 (6.2%)
Clinical presentation	
* Tinea capitis*	110 (84.6%)
*Kerion celsi*	20 (15.4%)
No animal contact	14 (10.8%)
Animal contact - Cat	35 (26.9%)
-Animal contact - Dog	81 (62.3%)
Negative direct mycological exam	68 (52.3%)
Positive direct mycological exam	62 (47.7)
Positive culture	94 (72.3%)
Culture Agents	
* Microsporum canis*	78 (60.0%)
* Microsporum gypseum*	3 (2.3%)
* Trichophyton mentagrophytes*	5 (3.8%)
* Trichophyton tonsurans*	1 (0.7%)
* Trichophyton rubrum*	3 (2.3%)
* Kodamaea ohmeri*	1 (0.7%)
Unidentified fungal species	3 (2.3%)
Negative culture	29 (22.3%)
Culture not performed	7 (5.4%)
Systemic treatment after evaluation	
Griseofulvin	122 (93.9%)
Fluconazole	4 (3.0%)
Terbinafine	2 (1.5%)
Itraconazole	2 (1.5%)

Data were analyzed using JMP® version 10.0. Normal continuous variables were expressed as mean ± standard deviation, and asymmetric variables as median and interquartile range (25^th^–75^th^ percentiles). Categorical variables were summarized by absolute and relative frequencies, with associations assessed by chi-square test. Statistical significance was set at p < 0.05.

The results are presented in Table 1. A total of 194 medical records were reviewed, of which 64 were excluded due to incomplete data. The mean duration of symptoms was 4.1-months. Animal contact was reported in 89.2% of patients. All patients presented with alopecia, scaling, and broken hairs.

Among cultures, 72.3% were positive, including 60.0% *Microsporum canis* (Fig. 1), 6.8% *Trichophyton spp.*, and 0.7% *Kodamaea ohmeri* (Fig. 2). Fig. 3 presents the distribution of agents identified in cultures.

**Figure 1:**
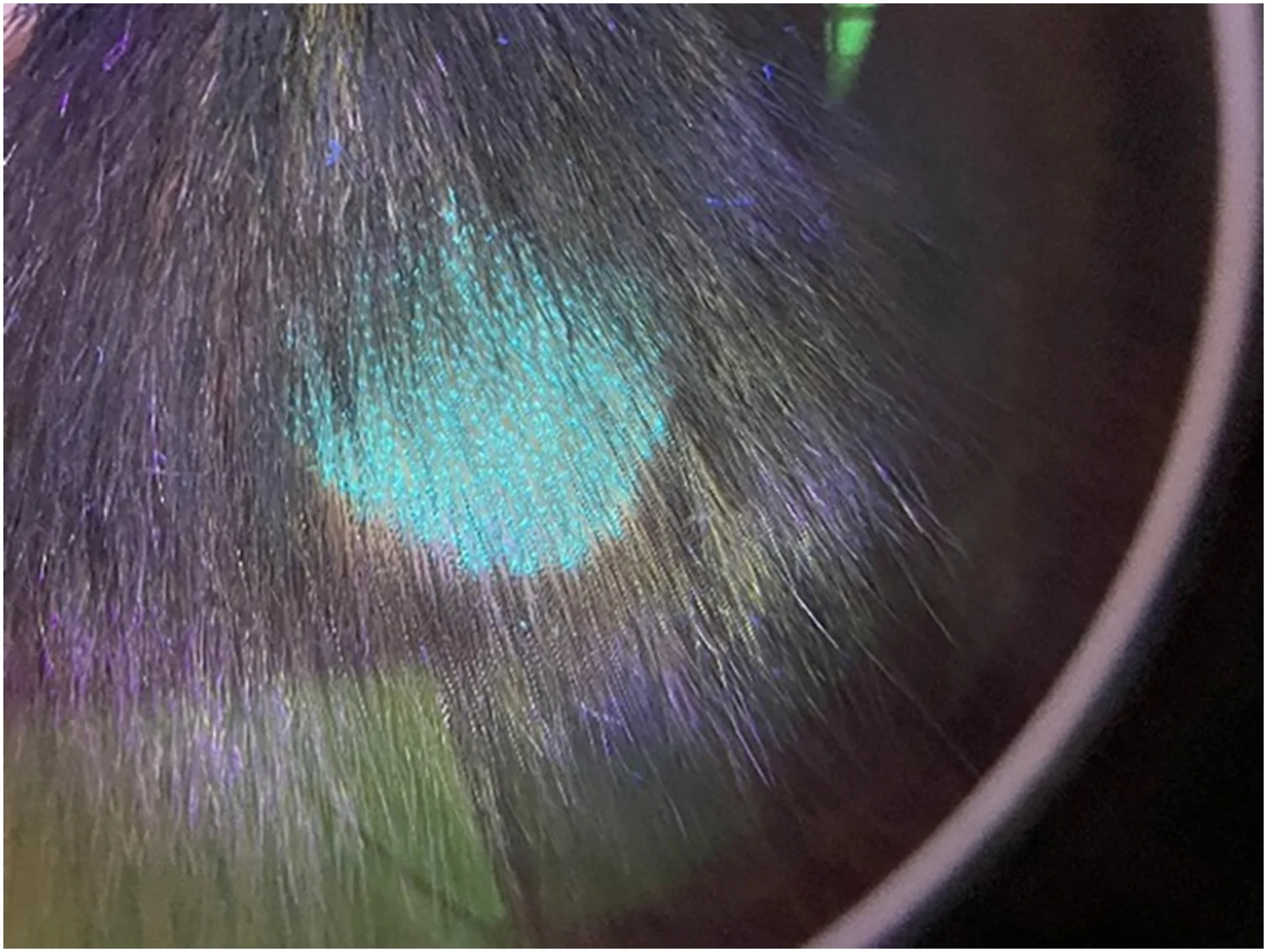
Round scaling area of alopecia, and blue fluorescence under Wood’s lamp, caused by *Microsporum canis*.

**Figure 2:**
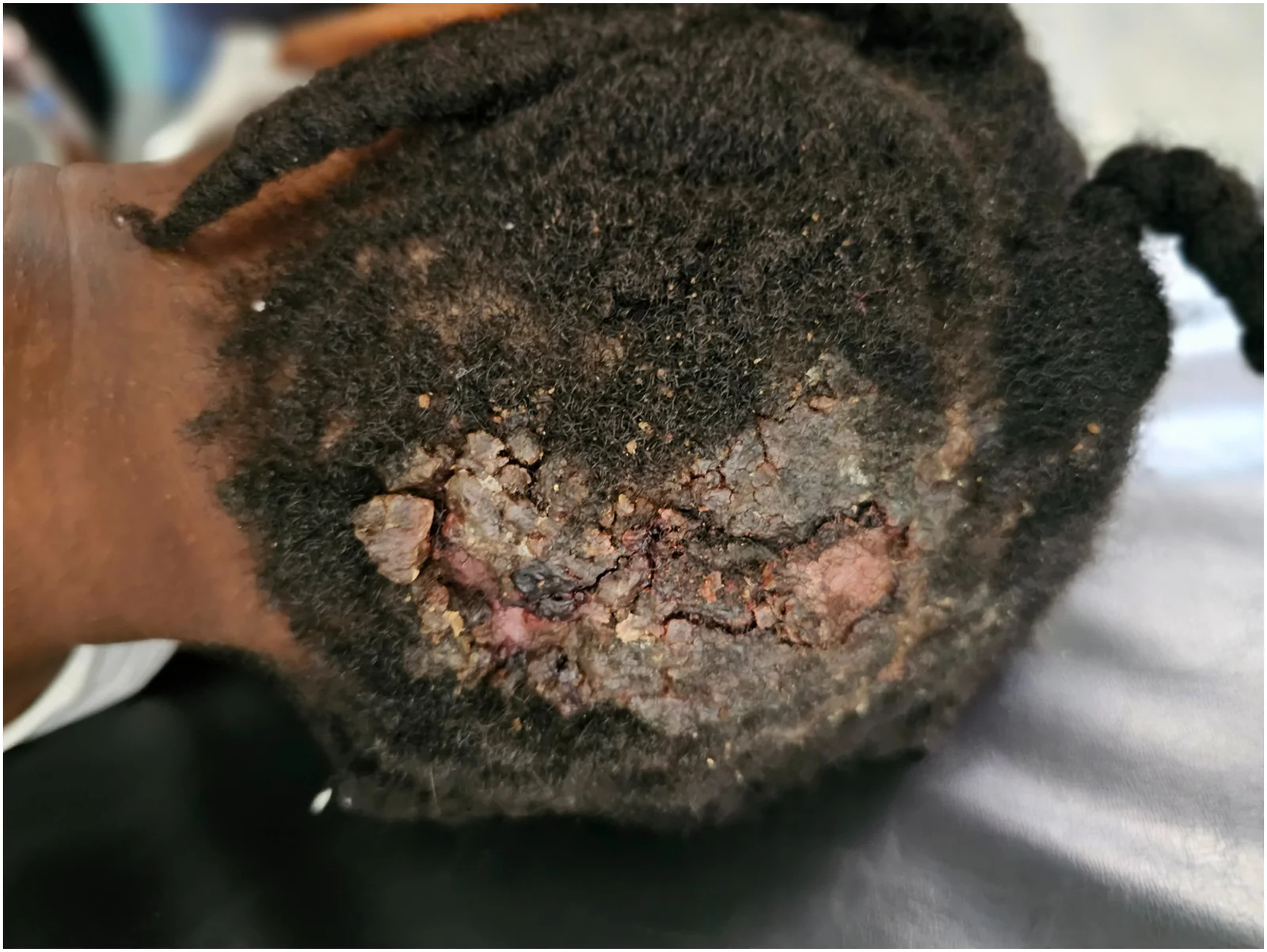
Plaque with adherent hemorrhagic and honey-colored crusts with areas of alopecia on the occipital region, caused by *Kodamaea ohmeri*.

**Figure 3:**
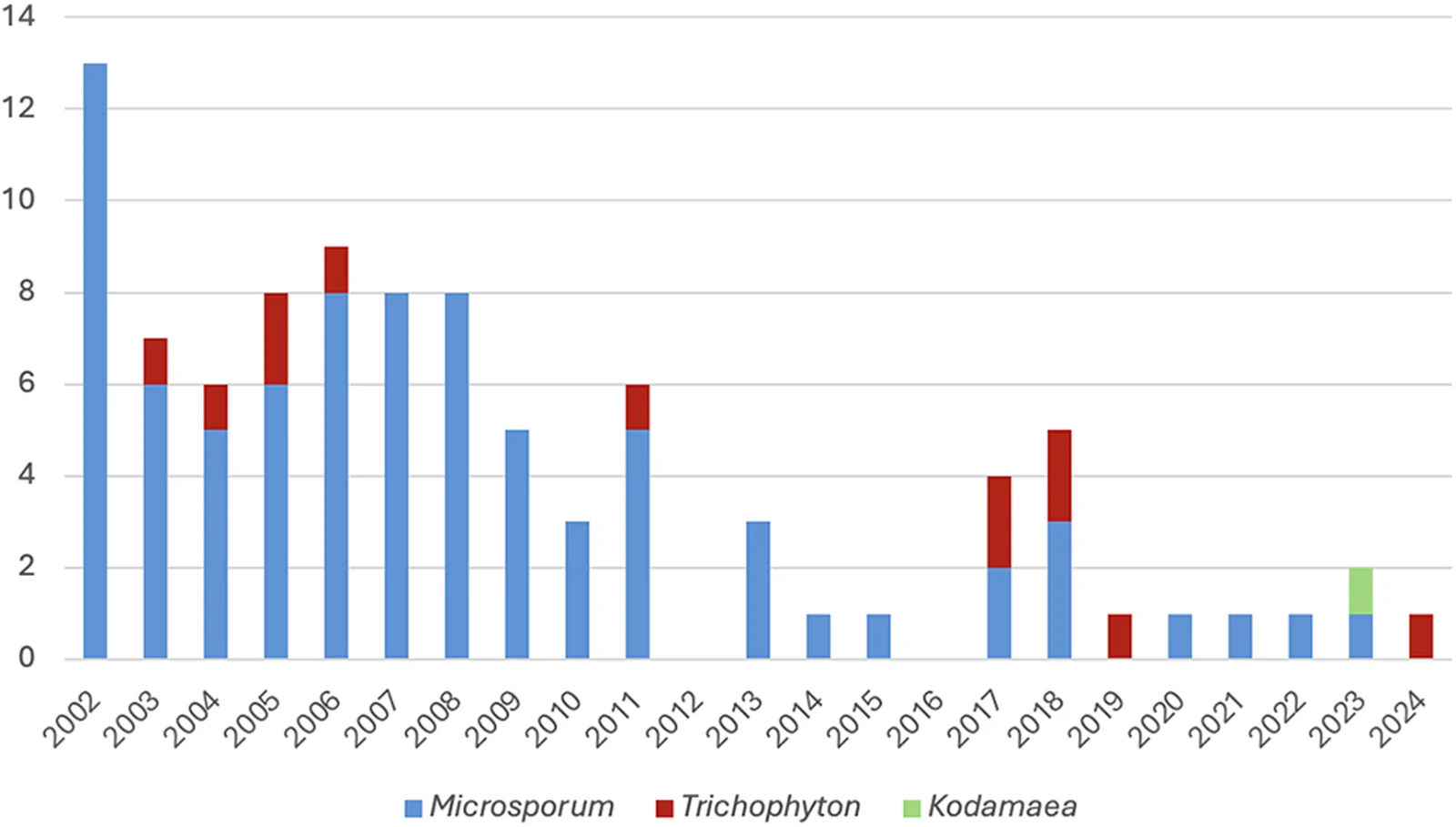
Distribution of the number of cases of each causal agent from 2002 to 2024. Note: Negative results or those in which culture was not performed were excluded. In 2012 and 2016, no cultures were positive.

Regarding the agent detected in cultures, during the first decade *Microsporum* predominated (92.1%), followed by *Trichophyton* (7.9%), whereas in the second decade there was a relative reduction in *Microsporum* (61.1%), a marked increase in *Trichophyton* (33.3%), and the emergence of *Kodamaea ohmeri* (5.6%) (p = 0.0013). After excluding the case of *Kodamaea ohmeri*, the difference remained statistically significant (p = 0.002), confirming that the shift was primarily due to variation between *Microsporum* and *Trichophyton* (Fig. 4).

**Figure 4:**
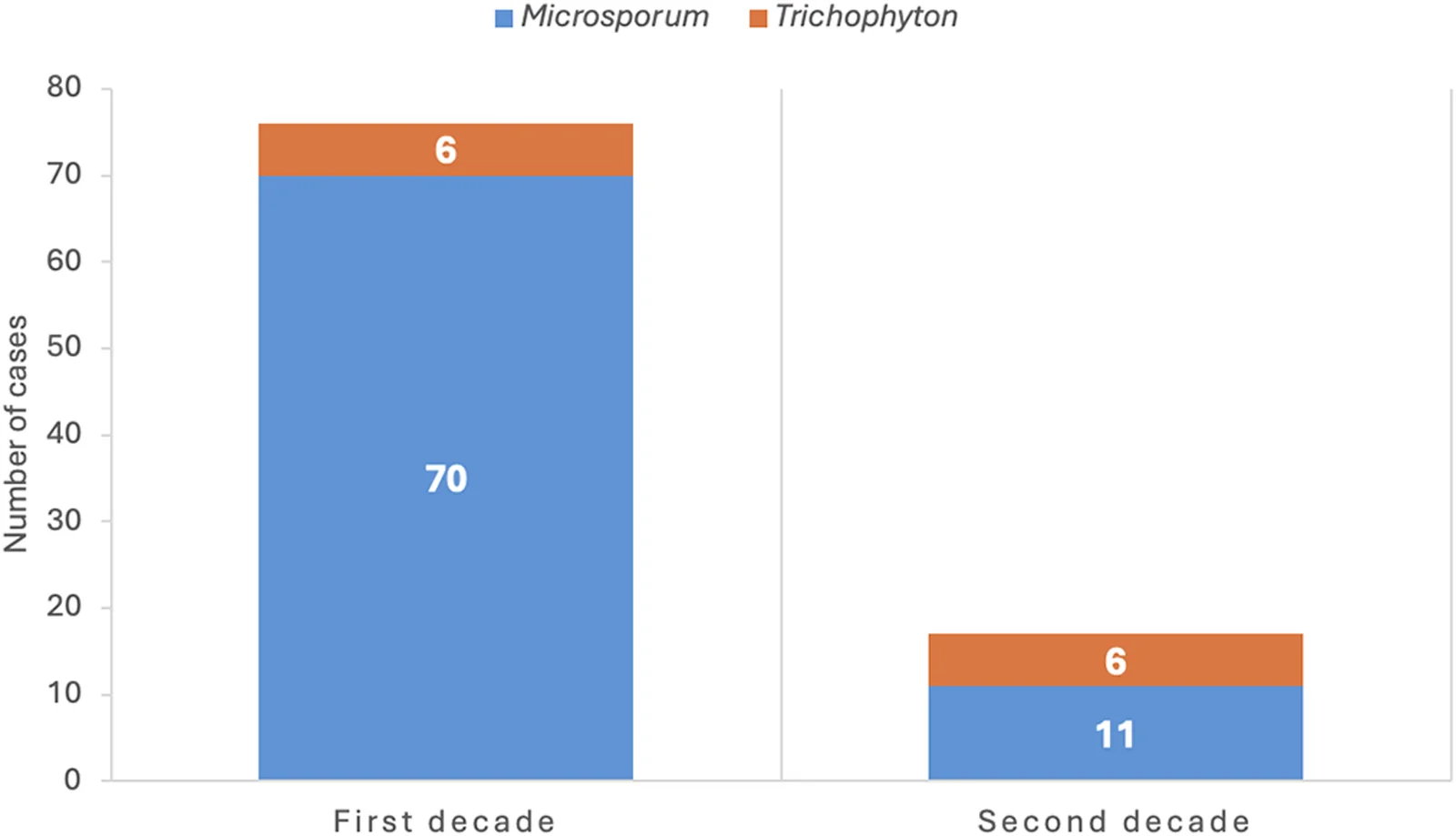
Distribution of etiological agents of *tinea capitis* by decade.

Systemic antifungal treatment was prescribed for 95.4% of patients. Griseofulvin was the most prescribed medication (99.9%) (Table 1). Seventy-two patients (55.4%) returned for follow-up evaluation, and all showed clinical improvement after 8 to 16 weeks of treatment.

In this 22-year analysis, *tinea capitis* predominated among school-aged boys. Although *Microsporum canis* was the most frequent agent, an epidemiological shift toward *Trichophyton* species was observed. Griseofulvin was the primary treatment, with clinical improvement noted in all returning patients. However, the 50% loss to follow-up, possibly due to early lesion resolution, precluded a comprehensive assessment of therapeutic response.

Shifts in dermatophyte species over time are linked to global migration, travel, and lifestyle changes.^3^, ^6^, ^7^ Regional variations are striking: *Trichophyton tonsurans* predominates in North America, while Europe has seen a significant rise in *Microsporum audouinii*. In parts of Asia, a transition from *Microsporum canis* to *Trichophyton* species is underway, whereas *Trichophyton rubrum* is increasing in Africa.^3^ These epidemiological shifts are critical for guiding regional therapeutic strategies.

In this study, *Microsporum canis* was the most frequent agent, confirming the predominance of zoophilic species in Southern Brazil, consistent with reports from the Southeast.^6^ This finding underscores the need to treat domestic animals, the reservoirs of the fungus, and to guide therapy and prevention strategies.^8^ However, a progressive change in the epidemiological profile was observed over the years, with an increase in *Trichophyton* and the unprecedented identification of *Kodamaea ohmeri*. This pattern follows global trends, in which European studies and recent reviews describe the gradual replacement of zoophilic species by anthropophilic ones, especially *Trichophyton tonsurans*, a phenomenon associated with factors such as urbanization, population migration, and habits of cohabitation with animals.^1^, ^2^, ^3^ In Brazil, the emergence of *Trichophyton* species is being observed, suggesting a similar transition process to that observed in other countries.^5^, ^7^

The isolation of *Kodamaea ohmeri*, although identified in one patient, deserves attention. In a systematic review, 51 cases of infection by *Kodamaea ohmeri* were described worldwide; of these, 12 occurred in the Americas, and no cases of *tinea capitis*. The presence of this agent may represent the emergence of infections caused by rare opportunistic yeasts in different regions.^9^

The treatment of choice for *tinea capitis* is systemic antifungal therapy, with griseofulvin and terbinafine.^3^, ^4^, ^10^ The choice of medication depends on the etiologic agent, and in practice treatment is initiated empirically before the fungal culture result. In the present study, 93.9% of participants used griseofulvin, and all had a good response at follow-up. A 2017 Cochrane review on systemic antifungal therapy for *tinea capitis* in children suggested that griseofulvin and terbinafine are effective treatments; however, complete cure was higher with griseofulvin in cases caused by *Microsporum* species and with terbinafine in those caused by *Trichophyton* species.^10^

Topical treatments for *tinea capitis* have a limited role, but are useful as adjuvant therapy, with the aim of eradicating viable spores and, consequently, reducing the risk of transmission, especially at the beginning of systemic treatment.^3^

During treatment, it is important to investigate and treat contact animals, which are the main source of transmission to humans when a zoophilic agent is suspected. For anthropophilic agents, control of fomites and appropriate hygiene are required.^8^

In the present study, there was a reduction in cases over the years, which may be related to improvements in personal and environmental hygiene, including adequate hair cleansing, use of clean personal items, and environments without humidity, which reduced fungal proliferation.^6^, ^7^, ^8^ Faster diagnostic methods allowed earlier initiation of treatment,^1^, ^3^ while awareness campaigns and zoonosis control measures reduced transmission of *Microsporum canis.*^1^, ^2^, ^6^, ^7^ In addition, changes in the epidemiological profile, with the emergence of *Trichophyton tonsurans*, a species with lower inflammatory impact and environmental persistence, also contributed to the decrease in severity of infections.^3^

Despite the limitations inherent to its retrospective design, this 22-year analysis revealed a relevant shift in the etiological profile of *tinea capitis* in Southern Brazil, with *Microsporum canis* remaining the predominant agent (60%), while a progressive decline in overall cases and a rise in *Trichophyton spp*. were observed ‒ mirroring global epidemiological trends.

## ORCID ID

Beatriz Carvalho: 0000-0002-5111-7327

Alon Coutinho Nascimento: 0009-0004-2633-0216

Talita Sana Valério Fontana: 0009-0003-9799-3773

Marjorie Uber: 0000-0003-2397-8047

Kerstin Taniguchi Abagge: 0000-0002-9824-2459

Renata Robl Imoto: 0000-0003-3726-5240

Vânia Oliveira de Carvalho: 0000-0002-4578-8781

## Research data availability

The entire dataset supporting the results of this study was published in this article.

## Financial support

None declared.

## Authors’ contributions

Esther Alves de Araujo Nunes: Data collection, analysis and interpretation; study conception and planning; preparation and writing of the manuscript; approval of the final version of the manuscript.

Mariana Aparecida Pasa Morgan: Study conception and planning; preparation and writing of the manuscript; manuscript critical review; approval of the final version of the manuscript.

Beatriz Carvalho: Data collection, analysis and interpretation; approval of the final version of the manuscript.

Alon Coutinho Nascimento: Data collection, analysis and interpretation; approval of the final version of the manuscript.

Talita Sana Valério Fontana: Data collection, analysis and interpretation; approval of the final version of the manuscript.

Marjorie Uber: Study conception and planning; approval of the final version of the manuscript.

Kerstin Taniguchi Abagge: Study conception and planning; approval of the final version of the manuscript.

Renata Robl Imoto: Study conception and planning; data collection, analysis and interpretation; manuscript critical review; approval of the final version of the manuscript.

Vânia Oliveira de Carvalho: Study conception and planning; data collection, analysis and interpretation; manuscript critical review; approval of the final version of the manuscript.

## Conflicts of interest

Esther Alves de Araujo Nunes, Mariana Aparecida Pasa Morgan, Beatriz Carvalho, Alon Coutinho Nascimento, Talita Sana Valério Fontana and Renata Robl Imoto have nothing to declare. Marjorie Uber has been a speaker for Kenvue and Mustela. Kerstin Taniguchi Abagge develops scientific materials and lectures for Leo Pharma, Theraskin, Expanscience, Kenvue and participates in clinical research with Sanofi-Genzyme. Vânia Oliveira de Carvalho has been an investigator for Sanofi Genzyme and a speaker for Expanscience, Kenvue, Galderma, Mantecorp, Megalabs and Royal Canin.
